# Effect of whole-body massage on growth and neurodevelopment in term healthy newborns: A systematic review

**DOI:** 10.7189/jogh.12.12005

**Published:** 2022-10-18

**Authors:** Mayank Priyadarshi, Vivek Kumar, Bharathi Balachander, Shuchita Gupta, Mari Jeeva Sankar

**Affiliations:** 1 Department of Neonatology, All India Institute of Medical Sciences, Rishikesh, Uttarakhand, India; 2 Department of Pediatrics, All India Institute of Medical Sciences, New Delhi, India; 3 Department of Neonatology, St. Johns Medical College Hospital, Bangalore, Karnataka, India; 4 World Health Organization, Geneva, Switzerland

## Abstract

**Background:**

Infant massage is commonly practiced in many parts of the world. However, the effectiveness of this intervention has not been reviewed for term, healthy newborns.

**Methods:**

This systematic review of randomized and quasi-randomized controlled trials assessed the effect of whole-body massage with or without oil, compared to no massage in term healthy newborns. Key outcomes were neonatal mortality, systemic infections, growth, behaviour (crying or fussing time, sleep duration), and neurodevelopment. We searched MEDLINE via PubMed, Cochrane CENTRAL, EMBASE, and CINAHL (updated till November 2021), and clinical trials databases and reference lists of retrieved articles. Two authors separately evaluated the risk of bias, extracted data, and synthesized effect estimates using mean difference (MD) and standardized mean difference (SMD). The GRADE approach was used to assess the certainty of evidence.

**Results:**

We included 31 randomized and quasi-randomized trials involving 3860 participants. Infant massage was performed by different care providers starting in the neonatal period and continuing for 1-2 months in most studies. Thirteen studies reported the use of oil with body massage. No study reported neonatal mortality or systemic infections. Meta-analyses suggested that whole-body massage may increase infant length at the end of the intervention period (median assessment age 6 weeks; mean difference (MD) = 1.6 cm, 95% confidence interval (CI) = 1.4 to 1.7 cm; low certainty evidence), but the effect on weight (MD = 340 g, 95% CI = 240 to 441 g), head circumference (MD = 0.8 cm, 95% CI = 0.6 to 1.1 cm), sleep duration (MD = 0.62 hours/d, 95% CI = 0.12 to 1.12 hours/d) and bilirubin levels (MD = -31.8 mmol/L or -1.8 mg/dL, 95% CI = -23.5 to -40.0 mmol/L) was uncertain. The effect on crying/fussing time at median 3 months of age, sleep duration at 6 months of age, weight, length, and head circumference at 6-12 months follow-up, and neurodevelopment outcomes, both at the end of the intervention period and follow-up was uncertain.

**Conclusions:**

Whole-body massage may improve the infant length at the end of the intervention period (median age 6 weeks, range 1-6 months) but the effect on other short- or long-term outcomes is uncertain. There is a need for further well-designed trials in future.

**Registration:**

Priyadarshi M, Balachander B, Rao S, Gupta S, Sankar MJ. Effect of body massage on growth and neurodevelopment in term healthy newborns: a systematic review. PROSPERO 2020 CRD42020177442.

Infant massage is commonly practiced in many parts of the world, especially in African and Asian countries. Systematic tactile stimulation of the body by hands is known as massage. Massage involves the process of rubbing and gentle slow stroking of body parts in turns, which can be done using different techniques. It can be done with or without the application of oil, such as mineral oil, olive oil, and other vegetable oils [1].

Several mechanisms may explain the positive effects of body massage on neonatal and infant outcomes. Body massage serves to improve circulation and soothe the peripheral and central nervous systems [2]. Massage has been shown to stimulate parasympathetic activity by acting on cutaneous pressure receptors and thereby increasing vagal activity [3]. Increased vagal activity leads to decreased cortisol and catecholamine levels as was demonstrated by Schanberg and colleagues [4]. Increased vagal activity also leads to an increase in bowel movements and hence, stool frequency, which reduces the enterohepatic circulation of bilirubin. This provides the rationale for the effect of massage on stress and jaundice, respectively. Secretion of insulin and gastrin is also enhanced with vagal activity, which may explain better absorption of food and growth. Massage has also been found to promote soothing behaviour in infants and better parent-infant interactions [5]. The tactile stimulation provided by the massage might contribute to a better neonatal experience that could help with overall development [2].

A 2013 Cochrane systematic review assessed the effect of massage on infants under 6 months of age [6]. The meta-analysis of 34 randomized controlled trials (RCTs) suggested that massage improves weight, length, and head circumference growth as well as developmental outcomes (gross motor skills, fine motor skills, personal and social behaviour), but most studies were found to be at high risk of bias. We did not find any systematic review evaluating the effectiveness of massage where the intervention had to be started in the neonatal period. The objective of the current review was to determine the effect of body massage with/without oil on critical neonatal and infant growth and development outcomes in term, healthy newborns.

## METHODS

Randomized controlled trials including cluster randomized trials or quasi-randomized trials in human neonates were eligible for this review. The study population was term healthy neonates up to 28 completed days after birth. Neonates who were low birth weight (BW) or preterm or had any illness or complications during birth hospitalization were excluded. The intervention was whole-body massage with or without oil started in the neonatal period and the comparator was no massage. The critical outcomes for this review were neonatal mortality (all-cause death in the first 28 days of life); systemic infections (sepsis, pneumonia, or possible serious bacterial infection); growth (weight, length, and head circumference, both short-term and long-term); neurodevelopment and neurobehavior (as assessed by standardized or validated tools); sleep characteristics (assessed by melatonin secretion, phase adjustment of rest-activity rhythms, duration of sleep, awakening episodes during sleep, the onset of sleep or based on paternal responses to a questionnaire) and parent-infant interactions (assessed by any standardized or validated tools or methods).

### Search methodology

Two review authors (MP and BB) independently searched MEDLINE (1966 onwards) via PubMed, Cochrane Central Register of Controlled Trials (CENTRAL, The Cochrane Library), EMBASE (1988 onwards), and CINAHL (1981 onwards). We conducted the first search till March 31, 2020. The search was then updated till November 30, 2021. Searches were limited to human studies. There were no language restrictions. Related conference proceedings (like Pediatric Academic Societies (PAS) abstracts) were also be searched for relevant abstracts. Organizations and researchers in the field were contacted, if necessary, for information on unpublished and ongoing trials. Reference lists of all relevant studies were searched. The clinical trial registry, www.clinicaltrials.gov was searched to identify any ongoing trial. The search strategy is provided in Appendix S1 in the **Online Supplementary Document**.

### Data extraction and management

Two review authors (MP and BB) extracted data independently using a pilot-tested data collection form to collect information on design, methods, participants, interventions, outcomes, and treatment effects from each included study. We discussed disagreements until we reached a consensus. If data from trial reports were insufficient, we contacted study authors to request further information or any clarifications, if required. Data was extracted from Cochrane review [6] for 17 studies as these were in Chinese language, and from abstract for one study as the full text was not available [7].

### Assessment of risk of bias in included studies

Two review authors (MP and VK) independently assessed the methodological quality of the selected studies. For randomized trials, quality assessment was undertaken using the Cochrane Risk of bias (RoB 2.0) tool [8]. Any disagreements between the review authors were resolved by discussion.

### Statistical analysis

Meta-analysis was performed using RevMan 5.4. For continuous variables, mean difference (MD) and weighted mean differences (WMD) or standardized MD (SMD) were calculated if outcomes were measured using different scales. We examined heterogeneity between trial results by inspecting the forest plots and quantifying the impact of heterogeneity using the *I^2^* statistic. If there was no significant heterogeneity (*I^2^*<60% or *P* ≥ 0.1, we pooled the results using the fixed-effect model. If there was significant heterogeneity (*I^2^*>60% or *P* < 0.1), we explored the possible causes of heterogeneity. If there was no obvious clinical heterogeneity, we used the random-effects model for meta-analysis. We used GRADEpro software for assigning the certainty of evidence [9].

## RESULTS

**Figure 1:**
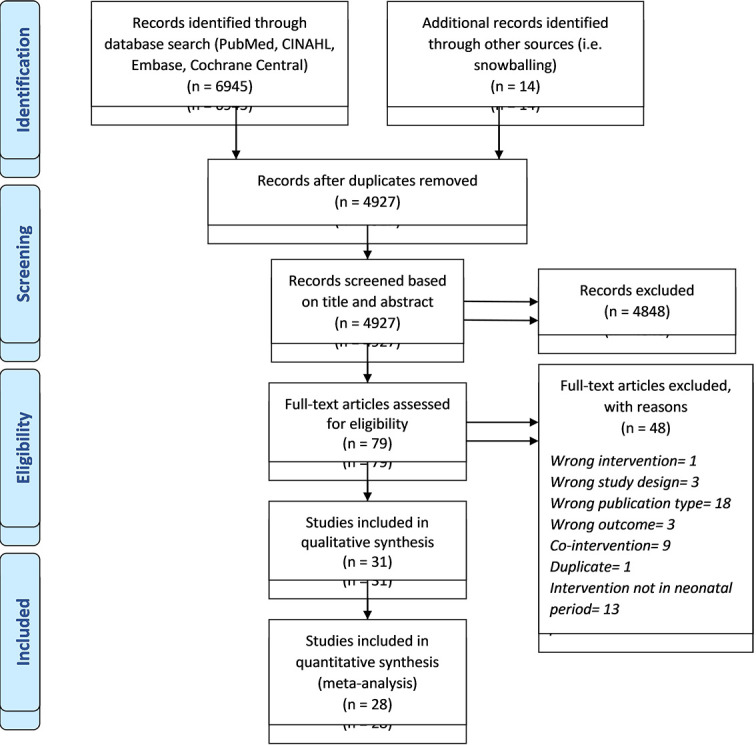
PRISMA flowchart depicting the selection of studies included in the review.

We included 31 studies, of which 28 studies were used for quantitative analysis (**Figure 1**). The characteristics of included studies are provided in **Table 1**.

**Table 1:** Characteristics of the studies included in the review

Study author, year	Setting	Study design	Study population/mean BW/gest	Intervention	Control	Outcomes of interest	Comments
Abedi 2017 [10]	Educational hospital, Iran; UMIC	RCT	Neonates with BW 2500-4000 g; birth through normal delivery; normal activity, appearance, and skin color; Apgar score above 7; and absence of any fetal abnormalities n = 66 (intervention = 33, control = 33), BW – 3222 g/, gest – 38.7 wk	Tactile-kinesthetic stimulation; 2 times a day, 15 min each time for 28 consecutive days; started within 24 h of age, provider: mothers (trained by authors) using olive oil	Routine care (no massage)	Weight, height, head circumference within 24 h of birth, on day 14 and day 28 (during home visits)	Non-supervised massage in the intervention group; no observation if control group neonates received massage
Chen 2011 [11]	Educational hospital, Japan; HIC	Quasi-RCT	Neonates with gest age 37-41 weeks, BW 2800-3600 g, Apgar score at the birth of 8-10 n = 69 (intervention = 29, control = 40), gest – 39.7 wk/BW – 3170 g.	Whole-body massage by specialized clinical staff for 15-20 min twice daily, 1 h after the morning and midday feed, from the first day to the fifth day postnatal, provider: specialized clinical staff using baby oil	Routine care (no massage)	Bilirubin (transcutaneous and serum) and stool frequency on days 1-5	Quasi-randomized design; very high rate of missing data (30%-45% in both groups due to neonates requiring phototherapy)
Cheng 2004 [12]	Hospital, China; UMIC	RCT	Newborn infants (population characteristics not available) n = 100 (intervention = 50, control = 50)	Massage for 15 min once daily for 42 d, provider: mothers	Routine care (no massage)	Head circumference, length, weight, sleep duration, and crying time on day 3 and day 42	Full text in Chinese, data retrieved from Cochrane review [6]
Dalili 2016 [13]	Valiasr hospital, Iran; UMIC	RCT	Neonates with gest 37-41 weeks, BW 2800-3600 g, Apgar score of 8-10, and no hemolytic condition n = 50 (intervention = 25, control = 25), gest – 38.2 wk/BW – 3113 g	Whole-body massage for 15-20 min three times a day from the first day to the fourth day, provider: mothers (trained by specialized trained midwives) using baby oil	Routine care (no massage)	Transcutaneous bilirubin (TcB) on day 4 and stool frequency from day 1-4	Baseline TcB on day 1 was not noted, therefore reduction could not be deducted
Ding 2005 [7]	Jiaozuo hospital, China; UMIC	RCT	Newborn infants (population characteristics not available) n = 300 (intervention = 150, control = 150)	Massage for 15 min, twice a day till 60 d, provider: professionals in hospital, and by parents or relatives at home	Routine nursing (no massage)	Sleeping time, arousal time, awakening times for 5 d, weight at birth and 42 d, milk intake at 10 and 60 d	Full-text not available, data from abstract
Duan 2002 [14]	Unclear setting, China; UMIC	RCT	Newborn infants (population characteristics not available) n = 160 (intervention = 80, control = 80)	Massage for 15 min per session 2 times per day for 42 d, provider: unclear	Routine care (no massage)	Weight, length, and head circumference at 42 d	Full text in Chinese, data retrieved from Cochrane review [6]
Elliot 2002 [15]	Community, Canada; HIC	RCT	Singleton, with a gest age of 40 weeks (±2 weeks), BW≥2500 g, a minimum Apgar score of 8 by five minutes, no extensive resuscitation, and no apparent congenital anomaly or significant birth injury n = 111 (group 1 = 31, group 2 = 29, group 3 = 24, control = 27), gest/BW – not mentioned	4 arm trial: group 1: massage for a minimum of 10 min daily, up to 20 min daily, 2 to 16 weeks of age, group 2: supplemental carrying group (carried infant in the carrier for a minimum of 3 h) group 3: combined intervention (1&2 both), provider: mothers trained by researchers using massage oil	Routine care (no massage)	Nursing Child Assessment Sleep Activity Record (NCASA) at 2, 6, 12, and 16 weeks, Nursing Child Assessment Feeding (NCAFS) and teaching at birth and 16 weeks, Early infant temperament questionnaire (EITQ) at 6 and 16 weeks, State-Trait Anxiety Inventory – STAI-T-anxiety scale, Parental sense of competence scale (PSOC) at 6 and 16 weeks, Difficult life circumstances scale (DLC)	Data presented only for group 1 and control (as supplemental carrying and combined intervention are not equivalent to massage)
Ferber 2002 [16]	Community, Israel; HIC	RCT	Dyads of mothers and full-term infants n = 21 (intervention = 13, control = 8), gest – 40wk/BW – 3.3-3.5 kg	30-min massage daily by the mother for 14 d, provider: mothers trained by researchers	Routine care (no massage)	Circadian rhythmicity at 6 and 8 weeks, excretion of the main melatonin metabolite 6-sulphatoxymelatonin at 6, 8, and 12 weeks	Small sample size, separate analyses of actigraph and hormonal data
Field 1996 [17]	Daycare nursery, USA; HIC	Quasi-RCT	Full-term newborn born to depressed mother n = 40 (intervention = 20, control = 20), gest – 39.4 wk/BW – 3483 g	15 min massage midway between morning feedings 2 d per week for 6 weeks. (with baby mineral oil), provider: researchers using mineral baby oil	Rocking sessions at midway between morning feedings for 15 min 2 d per week over 6 weeks for 12 sessions.	Sleep-wake behavior, temperament rating – colorado child temperament scale, salivary cortisol (ng/mL) on the first and last days of the study, weight and formula intake daily	Quasi-randomized design; active control group (rocking) but no tactile or kinesthetic stimulation
Field 2017 [18]	Educational hospital, USA; HIC	RCT	Primiparity and singleton birth and non-drug-exposed newborns, n = 76 BW – 3310 g/gest – 38.8 wk	Three-arm trial: intervention 1: lotion massage (lavender-scented massage lotion) intervention 2: no lotion massage (massage daily for 15 min before nighttime sleep for the one month, initiated within 24 h of age), provider: mothers trained by researchers	No massage	Sleep latency (min), night wakings, time asleep (hours), difficulty falling asleep (rating), problem sleeping (rating), confidence re-sleeping (rating) on day 1 and last day of the study	Number of participants not mentioned in individual groups, hence could not be used for meta-analysis
Gultom 2019 [19]	Community, Indonesia, LIC	Quasi-experimental study	Mothers (enrolled during pregnancy) and their newborns, n = 34 (intervention = 17, control = 17), BW – 3310 g/gest – not mentioned	25 min massage twice a day for 30-d; started soon after birth, provider: trained mothers using baby oil	No training provided to mothers; usual care	Weight, length, upper arm circumference; suckling frequency and duration at baseline and after 30 d of intervention	Quasi-experimental design; control group may have applied massage to their infants; blinding of assessors not clear
Gurol 2012 [20]	Obstetricclinic, Turkey; UMIC	Quasi-RCT	Neonates with a BW of 2600-4000 g, born at 38-42 gest week, 1st and 5th minute Apgar scores >7, and singleton pregnancy n = 120 (intervention = 60, control = 60), gest/BW – not mentioned	15-min whole-body massage sessions every day for 38 d; started within 5-7 d of life, provider: mothers trained by researchers using baby oil	Routine care (no massage)	Mother baby attachment using Maternal Attachment Inventory (MAI) score after 1 mo of intervention	Quasi-randomized design; control group may have applied massage to their infants
Inal 2012 [21]	Educational hospital, Turkey; UMIC	RCT	Newborns delivered at 38-40 weeks, healthy, BW between 10-90th centile, Apgar score above 7 at 1 and 5 min n = 145 (intervention = 76, control = 69), BW – 3362 g	Massage for 15 min daily till 6 mo age; started within 48 h of age, provider: mothers trained by researchers using baby oil	Routine care (no massage)	Lingual-cognitive, fine motor, gross motor, social skill self-care, and overall development score (Ankara Development Screening Inventory (ADSI) score) at 3 and 6 mo	Unacceptable attrition rate (30%) in both groups; no information on compliance rates
Jing 2007 [22]	Community, China; UMIC	RCT	Newborn infants (inclusion or exclusion criteria not specified) n = 180 (intervention = 90, control = 90), BW – 3.3 kg	Procedure performed 1-2 times per day, massage for 15 min per session, and motion training for 5 min per session, from birth to 6 mo of age and massage 8 min, motion training 12 min after 6 mo of age, Provider: trained parents	Routine care (no massage)	Weight (kg) and length (cm) at 0, 1, 6 and 12 mo; developmental quotient (Gessell Developmental Schedule) at 1, 6 and 12 mo	Motion training is included in the Johnson massage method, therefore the whole procedure was considered as massage
Ke 2001 [23]	Unclear setting, China; UMIC	RCT	Newborn infants (population characteristics not available) n = 400 (intervention = 200, control = 200)	Fifteen minutes of massage three times a day for 42 d plus an additional method of kneading the back, provider: unclear	Routine care (no massage)	Weight, length, and head circumference at 42 d	Full text in Chinese, data retrieved from Cochrane review [6]
Koniak-griffin 1988 [24]	Community hospital, USA; HIC	RCT	Uncomplicated full term pregnancies ending with a healthy neonate n = 81 (group 1 = 20, group 2 = 20, group 3 = 21, control = 20), gest/BW – not mentioned	4 arm trial: Group 1: nnimodal (massage); 5-7 min daily for 3 mo group 2: multimodal (placement in a hammock with multisensory stimulation) group 3: combined stimulation (1&2 both),provider: mothers trained by researchers	Routine care (no massage)	Weight, Bayley Scales of Infant Development (BSID), Nursing Child Assessment Teaching Scale (NCATS), Revised Infant Temperament Questionnaire (RITQ), Eyberg’s Child behavior Inventory at 4, 8, and 24 mo	Data presented only for group 1 and control (as multimodal stimulation and combined stimulation are not equivalent to massage)
Liu C 2001 [25]	Community, China; UMIC	RCT	Infants 0-2 mo (population characteristics not available) n = 232 (intervention = 159, control = 73)	Massage 2 -3 times daily for 15 min for at least 3 mo, provider: mothers trained by researchers	Routine care (no massage)	Bayley's mental and psychomotor development index, sleep habits, growth (height, weight, head circumference, chest circumference) at 6 mo	Full text in Chinese, data retrieved from Cochrane review [6]
Liu CL 2005 [26]	Unclear setting, China; UMIC	RCT	Newborn infants (population characteristics not available) n = 80 (intervention = 40, control = 40)	15 min of massage twice daily over 42 d, provider: unclear	Routine care (no massage)	Weight at 42 d	Full text in Chinese, data retrieved from Cochrane review [6]
Liu DY 2005 [27]	Unclear setting, China; UMIC	RCT	Newborn infants (population characteristics not available) n = 200 (intervention = 100, control = 100)	15 min of massage twice daily over 42 d, provider: nurses	Routine care (no massage)	Weight, height, head circumference, and length of sleep at 42 d	Full text in Chinese, data retrieved from Cochrane review [6]
Maimaiti 2007 [28]	Community, China; UMIC	RCT	Newborn infants (inclusion or exclusion criteria not specified) n = 200 (intervention = 100, control = 100), gest – 38 wk/BW – 3.4 kg	Massage 3 times daily (duration: unclear), provider: professionals initially then trained parents	Routine care (no massage)	Infant physical development characteristics include the angle at which the infant can rise from a prone position, sight and auditory tracking, and the ability to smile	Full text in Chinese, data retrieved from Cochrane review [6]
Na 2005 [29]	Unclear setting, China; UMIC	RCT	Newborn infants (population characteristics not available) n = 80 (intervention = 40, control = 40)	15 min of massage three times daily for 28 d, provider: unclear	Routine care (no massage)	Weight, height, head circumference at 28 d	Full text in Chinese, data retrieved from Cochrane review [6]
Seyyedrasooli 2014 [30]	Tabriz al-Zahra hospital, Iran; UMIC	RCT	First-day skin bilirubin level less than 5mg/dl, breastfed neonates n = 50 (intervention = 25, control = 25), gest – 38.2 wk/BW – 3113 g	Moderate pressure massage for four days (day 1-4); three sessions a day, 15 min per session, provider: professionals initially then trained mothers	Routine care (no massage)	Transcutaneous bilirubin (TcB) and stool frequency from day 1-4	Unacceptable attrition rate (30%) in the intervention group
Shao 2005 [31]	Unclear setting, China; UMIC	Quasi-RCT	Newborn infants (population characteristics not available) n = 210 (intervention = 105, control = 105)	15 min of massage twice daily over 30 d, provider: unclear	Routine care (no massage)	Weight at 30 d	Full text in Chinese, data retrieved from Cochrane review [6]; could not be used for meta-analysis
Shi 2002 [32]	Unclear setting, China; UMIC	RCT	Newborn infants (population characteristics not available) n = 80 (intervention = 40, control = 40)	15 min of massage twice daily over 28 d, provider: unclear	Routine care (no massage)	Weight and height at 28 d	Full text in Chinese, data retrieved from Cochrane review [6]
Sun 2004 [33]	Unclear setting, China; UMIC	RCT	Population characteristics not available n = 210 (intervention = 105, control = 105)	Massage for 15 min 2 times per day for 42 d, provider: unclear	Routine care (no massage)	Weight (post-intervention), bilirubin (day 7), sleeping time	Full text in Chinese, data retrieved from Cochrane review [6]
Wang 1999 [34]	Unclear setting, China; UMIC	RCT	Newborn infants (population characteristics not available) n = 60 (intervention = 30, control = 30)	15 min of massage three times daily over 42 d, provider: unclear	Routine care (no massage)	Weight at 42 d	Full text in Chinese, data retrieved from Cochrane review [6]
Wang 2001 [35]	Community, China; UMIC	RCT	Newborn infants (population characteristics not available) n = 57 (intervention = 27, control = 30)	Massage for 15-20 min per day for 2 mo, provider: trained professionals then mothers	Routine care (no massage)	0-3 education development checklist (Capital Institute of Children 0-3 y old checklist), weight at 60 d	Full text in Chinese, data retrieved from Cochrane review [6]
Xua 2004 [36]	Unclear setting, China; UMIC	RCT	Newborn infants (population characteristics not available) n = 124 (intervention = 61, control = 63)	15-20 min of massage twice daily over three months, provider: unclear	Routine care (no massage)	Duration of sleep; frequency of night wakes and crying; length of crying; length of time for a normal sleeping pattern at 3 and 6 mo	Full text in Chinese, data retrieved from Cochrane review [6]
Ye 2004 [37]	Unclear setting, China; UMIC	RCT	Newborn infants (population characteristics not available) n = 100 (intervention = 50, control = 50)	10-15 min of massage twice daily over 42 d, provider: unclear	Routine care (no massage)	Weight at 42 d	Full text in Chinese, data retrieved from Cochrane review [6]
Zhai 2001 [38]	Unclear setting, China; UMIC	Quasi-RCT	Newborn infants (population characteristics not available) n = 100 (intervention = 50, control = 50)	15 min of massage three times daily over 30 d, provider: unclear	Routine care (no massage)	Weight, height, and head circumference at 30 d	Full text in Chinese, data retrieved from Cochrane review [6]; could not be used for meta-analysis
Zhu 2010 [39]	Community, China; UMIC	Quasi-RCT	Newborn infants (population characteristics not available) n = 115 (intervention = 55, control = 60)	Massage for 15-20 min per session, 2-3 times a day for 3 mo, provider: parents	Routine care (no massage)	Neonatal behavioral neurological assessment score (NBNA) at 1 mo; mental development index (MDI) and psychomotor development index (PDI) using adapted China Institute of Psychology and Child Development Center scales at 3 mo, head circumference measurements at 6 mo	Full text in Chinese, data retrieved from Cochrane review [6]

HIC – high-income country, LIC – low-income country, RCT – randomized controlled trial, UMIC – upper middle-income country, BW – birth weight, Gest – gestation

### Design

Twenty-four of the 31 included studies were randomized and seven were quasi-randomized trials. In the seven quasi-randomized studies, randomization was done based on birth date or time, or admission number in six studies and was not described in one study [19]. All included studies were two arm-trials except one which was a 3-arm [18] and two which were 4-arm trials [15,24]. For the multi-arm trials, we used data from “massage only” and control or no massage groups.

#### Setting

Six studies were done in high-income countries (USA, Canada, Japan, and Israel), 24 studies in upper-middle-income countries (China, Turkey, and Iran), and one in a low-income country (Indonesia). Three studies were primarily hospital-based, six were conducted in both hospitals and community settings after discharge, and nine trials were community-based. One study enrolled and followed up neonates in daycare nurseries [17]. Twelve studies, conducted in China, did not specify the setting.

### Participants

The participants were term neonates, with normal BW and no major comorbidities (asphyxia, anomalies, etc.). One study included neonates born to depressed mothers but the neonates were clinically healthy [17]. Though population characteristics were not mentioned in 17 studies (2577 neonates) from China, there was no indication that the included neonates had any illness or complications. In one study, the participants were infants aged 0-2 months, and mean age at enrolment or proportion of neonates in the enrolled population were not specified, but we included this study assuming at least half of the enrolled participants to be neonates [25].

### Intervention

The intervention was initiated from birth in two trials [19,22], within 24 hours of birth in six trials, within 48 hours of birth in one trial [21], after five days of birth in one trial [20] and after the second week of life in one trial [15]. The exact timing of initiation was not reported in 20 trials but specified that the participants were newborns (19 trials) or 0-2-month infants [25].

There was variation in massage techniques across studies, but all included studies applied whole-body massage, with or without the use of oil. Four trials employed the massage technique promoted by Johnson and Johnson [40]. Four trials applied the method prescribed by Field in 1986 [11,13,17,18,41]. One trial had an additional component of kneading the back along with whole-body massage [23]. Most studies from China did not describe the massage technique.

Thirteen trials used oils or emollients for massage but only nine specified the type of oil used. Six studies used “baby oil” or “massage oil” with no other description, one study each used olive oil [10], mineral oil [17], and lavender-scented lotion [18]. In the rest four trials, methods suggest that some kind of oil was used but the description is not provided. The remaining 18 trials do not specify the use of oils or other emollients during the massage.

Duration and dosage of intervention were variable across included trials: 4 days, 15-20-minute sessions, 2-3 times daily (3 trials); 14 days, 30-minute sessions, once daily (1 trial); 28 days, 15-minute sessions, 2-3 times daily (7 trials); 6 weeks, 15-minute sessions, 1-3 times daily (10 trials); 2 months, 15-20-minute sessions, 1-2 times daily (2 trials); 14 weeks, 10-20-minute sessions, once daily (1 trial); 3 months, 15-20-minute sessions, twice daily (4 trials); 6 months, 15-minute sessions, once daily (1 trial); and 12 months, 15-minute sessions once daily (1 trial). One trial did not specify the duration of the intervention [28].

In two studies [11,17], researchers provided massage while in other studies, massage was provided by their care providers (mothers) after training. Eleven studies from China did not mention the specifics of massage providers in their methods [14,23,26,29,31-34,36-38].

All studies had a “no massage” comparator group. One study used “rocking” as a comparator [17]. Since rocking is considered a normal soothing technique for neonates, this study was included in the review. Routine newborn care was provided by all studies.

### Risk of bias in included studies

A summary of risk of bias assessment in the included studies is provided in Appendix S2 in the **Online Supplementary Document**. Thirty of the 31 trials were judged to be at high risk of bias, with most studies being at high risk of bias in the randomization process, either due to non-reporting of random sequence generation or allocation concealment or owing to their quasi-randomized design.

### Effects of interventions

Neonatal and infant mortality, systemic infections (sepsis, pneumonia, or possible serious bacterial infection), and adverse events were not reported by any of the included studies. (Table S1 in the **Online Supplementary Document**).

### Infant growth

The mean weight, length, and head circumference of infants were reported at two time points. First, at the end of the intervention period for which the median age at assessment was 6 weeks, ranging from 1 to 6 months for length and weight, and 1 to 3 months for head circumference. The second assessment was conducted at follow-up which varied for different outcomes and is reported below.

**Figure 2:**
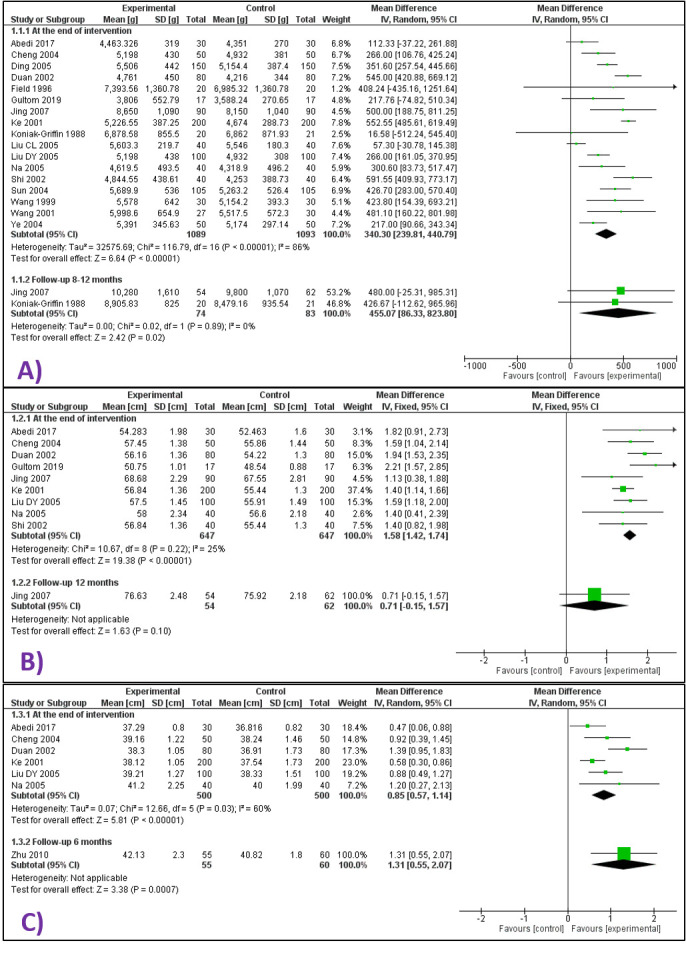
Forest plots for anthropometric outcomes. **Panel A.** Forest plot for comparison – massage vs no massage Outcome – infant weight at i) the end of intervention period and at ii) 8-12 months follow-up. **Panel B**. Forest plot for comparison – massage vs no massage, Outcome – infant length at i) the end of intervention period and at ii) 12 months follow-up. **Panel C**. Forest plot for comparison – massage vs no massage, Outcome – infant head circumference at i) the end of intervention period and at ii) 6 months follow-up.

The mean length of infants in the massage group was higher at the end of the intervention period (MD = 1.6 cm higher, 95% confidence interval (CI) = 1.4 cm higher to 1.7 cm higher; 9 studies, 1294 participants; low certainty evidence) (**Figure 2** Panel B). Funnel plot did not reveal any visual asymmetry (Figure S3 Panel B in the **Online Supplementary Document**). There was little data on infant length at 12 months follow-up (MD = 0.7 cm, 95% CI = 0.2 cm lower to 1.6 cm higher; 1 study, 116 participants; very low certainty evidence).

The mean weight of infants receiving whole-body massage was higher at the end of the intervention period (MD = 340 g higher, 95% CI = 240 g higher to 441 g higher; 17 studies, 2182 infants; very low certainty evidence), but there was little data on infant weight at 8-12 months follow-up (MD = 455 g higher, 95% CI = 86 g higher to 824 g higher; 2 trials, 157 infants; very low certainty evidence) (**Figure 2** Panel A). There was no visual asymmetry on funnel plot (Figure S3 Panel A in the **Online Supplementary Document**).

The mean head circumference of infants in the massage group was higher at the end of the intervention period (0.8 cm higher, 95% CI = 0.6 cm higher to 1.1 cm higher; 6 studies, 1000 participants; very low certainty evidence), with little data at 6 months (MD = 1.3 cm higher, 95% CI = 0.6 cm higher to 2.1 cm higher; 1 study, 115 participants; very low certainty evidence) (**Figure 2** Panel C).

### Infant behaviour

**Figure 3:**
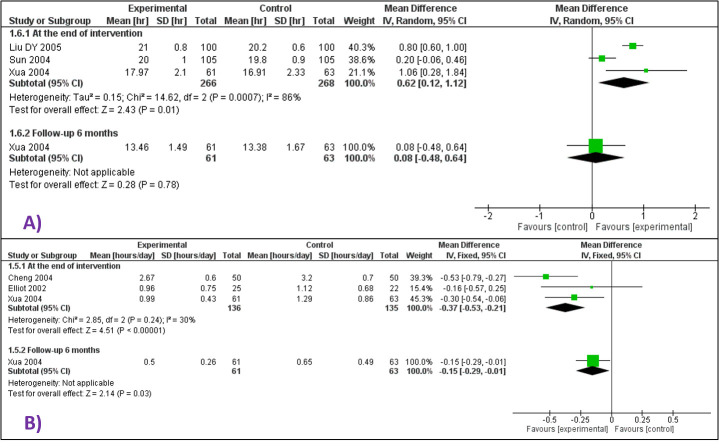
Forest plots for behavioural outcomes. **Panel A.** Forest plot for comparison – massage vs no massage. Outcome – sleep duration at i) the end of intervention period and at ii) 6 months follow-up. **Panel B.** Forest plot for comparison – massage vs no massage. Outcome – crying or fussing time at i) the end of intervention period and at ii) 6 months follow-up.

The mean sleep duration was slightly higher at the end of the intervention period (median age of 6 weeks; range 6 weeks to 3 months) for infants receiving whole-body massage (MD = 0.62 hours/d higher, 95% CI = 0.12 hours/d higher to 1.12 hours/d higher; 3 studies, 534 participants; very low certainty evidence), but there was little data at 6 months of age (MD = 0.08 hours/d higher, 95% CI = 0.48 hours/d lower to 0.64 hours/d higher; 1 study, 124 participants; very low certainty evidence) (**Figure 3** Panel A).

There was little data for mean crying or fussing time for infants at a median age of 3 months (range 6 weeks to 4 months) (MD = 0.36 hours/d lower, 95% CI = 0.16 hours/d lower to 0.53 hours/d lower; 3 studies, 271 participants; very low certainty evidence), and at 6 months of age (MD = 0.15 hours/d lower after receiving massage till 3 months of age; 95% CI = 0.01 hours/d lower to 0.29 hours/d; 1 study, 124 participants; very low certainty evidence) (**Figure 3** Panel B).

There was little data for maternal-infant interaction measured using Maternal Attachment Inventory (MAI) at the age of 6 weeks (MAI mean score, MD = 5.77 higher, 95% CI = 0.95 higher to 10.59 higher; 1 study; 117 participants; very low certainty evidence).

### Neurodevelopment outcomes

Three studies reported Psychomotor Development Indices (PDI) at the end of the intervention period (median age of 4 months)- two studies measured PDI using the Bayley Scale of Infant Development [24,25] and one study used the Levin PDI tool adapted by the China Institute of Psychology and Child Development Center [39]. Only one study reported PDI at 24 months of age after providing intervention for 3 months after birth [24].

**Figure 4:**
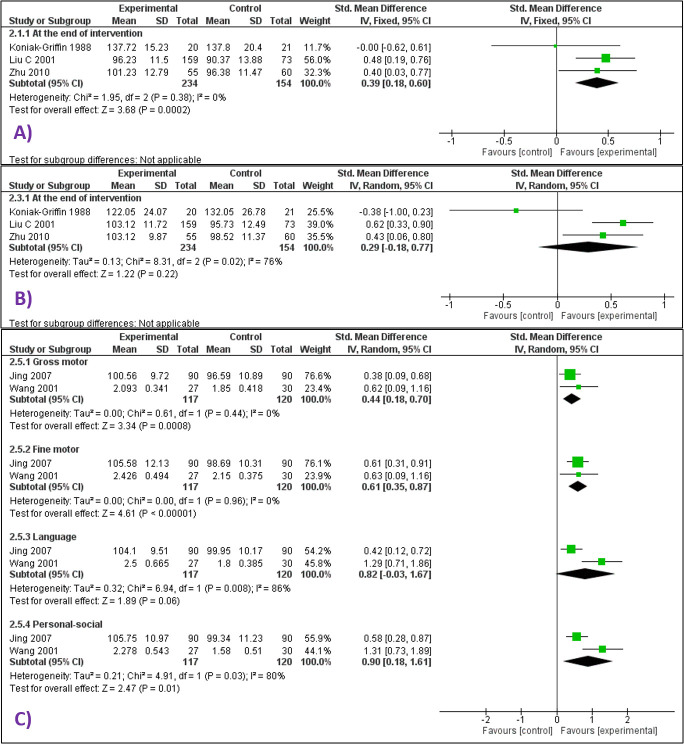
Forest plots for neurodevelopment outcomes. **Panel A.** Forest plot for comparison – massage vs no massage. Outcome – psychomotor development indices (PDI) at the end of the intervention period (median age 4 months). **Panel B**. Forest plot for comparison – massage vs no massage. Outcome – mental development indices (MDI) at the end of the intervention period (median age 4 months). **Panel C**. Forest plot for comparison – massage vs no massage. Outcome – gross motor, fine motor, language and personal-social development.

There was limited data for mean PDI scores at the end of the intervention period (SMD 0.39 SD higher, 95% CI = 0.18 higher to 0.6 higher; 3 studies, 388 infants; very low certainty evidence) (**Figure 4** Panel A), and at 24 months after receiving massage till 3 months of age (MD = 7.52 higher, 95% CI = 1.49 lower to 16.53 higher; 1 study, 41 infants; very low certainty evidence).

Similarly, there was little data on Mental Development Indices (MDI) at the end of the intervention period (SMD = 0.29 SD higher, 95% CI = 0.18 lower to 0.77 higher; 3 studies and 388 participants; very low certainty evidence) (**Figure 4** Panel B) and at 24 months (MD = 8.59 higher, 95% CI = 1.62 lower to 18.8 higher; 1 study, 41 participants; very low certainty evidence).

One study assessed gross motor, fine motor, language, and personal-social development at the age of 6 months using Gessel Developmental Quotient [22] and one study at the end of the 2-month intervention period using the Capital Institute Mental checklist [35]. Given the similarity in the domains of these tools, we meta-analyzed the outcomes but still there was little data (gross motor skills, SMD = 0.44 SD higher, 95% CI = 0.18 higher to 0.7 higher; fine motor skills, SMD = 0.61 SD higher, 95% CI = 0.35 higher to 0.87 higher; personal-social behaviour, SMD = 0.9 SD higher, 95% CI = 0.18 higher to 1.61 higher; language, SMD = 0.82 SD higher, 95% CI = 0.03 lower to 1.67 higher (very low certainty evidence) (**Figure 4** Panel C).

There was limited data for development outcomes at 12 months (fine motor skills, MD = 8.12 higher, 95% CI = 4.57 to 11.67; language MD = 7.9 higher, 95% CI = 4.1 to 11.7; personal-social behaviour, MD = 6.19 higher, 95% CI = 2.55 to 9.83; and gross motor skills, MD = 2.85 higher, 95% CI = -2.48,8.18 (very low certainty evidence).

### Additional outcome

**Figure 5:**
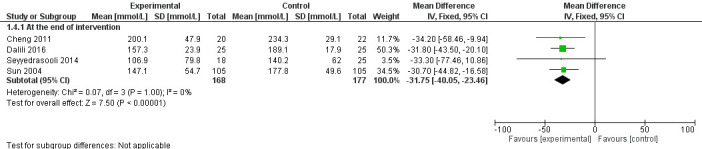
Forest plot for comparison – massage vs no massage. Outcome – bilirubin levels.

The mean bilirubin levels were slightly lower in the massage group at the median age of 4 days (MD = 31.75 mmol/L lower, 95% CI = 23.46 mmol/L lower to 40.05 mmol/L lower; 4 studies, 345 participants; very low certainty evidence) (**Figure 5**).

### Results from studies with qualitative data

Three studies could not be included in quantitative analysis [18,31,38]. One study employed a three-arm trial, comparing the effects of “lotion” massage, “no lotion” massage, and “no” massage on sleep behaviour in 76 mothers and their infants [18]. At the end of 1 month, the lotion massage group showed a shorter latency to sleep and longer sleep duration for mothers and fewer night awakenings and longer sleep duration for their infants, compared with the other two groups. However, the authors did not mention the number of participants in each group. One trial examined the effect of massage vs no massage on the weight of 210 neonates at the end of 30 days and found a significantly higher weight gain in the massage group (only means and significance levels provided) [31]. Another trial compared the massage group with no massage group, looking at the effects on growth (weight, length, and head circumference) at the end of 1 month in 100 neonates [38]. There was significantly better growth in the massage group, though the study provided means and significance levels only.

## DISCUSSION

Our review suggests that whole-body massage may increase infant length at the end of the intervention period (median assessment age 6 weeks, range 1-6 months), but the effect on weight, head circumference, sleep duration, and bilirubin levels at the end of the intervention period was uncertain. The effect on crying/fussing time at median 3 months of age, sleep duration at 6 months of age, weight, length, and head circumference at 6-12 months follow-up, and neurodevelopment outcomes, both at the end of the intervention period and follow-up was also uncertain. None of the studies reported neonatal and infant mortality, systemic infections (sepsis, pneumonia, or possible serious bacterial infection), and adverse events.

Though limited by low quality evidence, whole-body massage may prove to be one of the interventions among others which have been shown to improve infant length during the initial months of life [42]. For all other outcomes, the direction of effect favored whole-body massage for various outcomes specified above, but the evidence was ascertained as very low certainty either because of the paucity of data resulting in wide confidence intervals or high risk of bias in most studies. The effects of intervention can be explained by several plausible biological mechanisms described earlier related to its effect on various body systems. However, the pooled effect sizes for several outcomes in this review are quite large in magnitude and it is difficult to explain these completely based on the alluded mechanisms [3,4]. There is a need to further understand the biological mechanisms that may underlie some of these potential benefits. The other possible reason for such results may be related to the lack of methodological robustness in the included trials. Thirty of the 31 included studies were at high risk of bias, mainly owing to bias in randomization, measurement and missing outcome domains. To explore the effect of measurement bias, we dropped the studies which were at high risk of bias for measurement domain and performed sensitivity analyses with the remaining studies, which yielded similar results as their primary analyses (Figure S4 Panels A-C in the **Online Supplementary Document**). The evidence was also downgraded for unexplained heterogeneity. Though the population and interventions in the included trials were consistent, clinical heterogeneity might have been the result of differences in the settings, massage providers, duration of intervention, massage techniques, and outcome assessment. Therefore, while it appears that the intervention is probably beneficial, there is a need for larger, well-designed studies addressing the short- and longer-term outcomes.

The findings of our review are similar to the Cochrane review published by Bennett et al, evaluating the impact of massage on healthy infants less than six months of age [6]. Of its 34 included trials, 22 trials overlapped with our review. The rest 12 trials were excluded from our review, either due to initiation of intervention in the post-neonatal period or due to the different nature of the intervention (multi-modal). Similar to our review, the review suggested significant positive effects on growth and neurodevelopment, but evidence being very low certainty for any meaningful conclusions. Another review evaluated the effect of massage on neonatal jaundice [43]. Two of the six included trials recruited healthy term neonates and were included in our review also, but the rest four were excluded as they enrolled “jaundiced” neonates who received massage as an adjunct to phototherapy. The review concluded that massage was effective in the reduction of serum and transcutaneous bilirubin levels in neonates. The findings are similar to our review, though we ascertained the evidence to be very low certainty due to a very serious risk of bias and serious imprecision. Similar reviews have been done to evaluate the effect of massage on feed intolerance, growth, and neurodevelopment in preterm neonates [44,45].

This review has tried to answer a research question that is important from both a clinical and a public health perspective. A rigorous methodology was followed to conduct this review, with an all-inclusive literature search and no language filters. Eighteen trials were available in the Chinese language (17 trials) and abstract form only (1 trial). Though we tried to translate these articles using a machine translation service (Google translate), however, for accuracy and completeness, we cross-checked our findings with the Cochrane review [6]. We did not include the China Knowledge Resource Integrated Database (CNKI) database as a part of our literature search, which contributed to most of the trials in the Cochrane review, mainly due to the non-availability of freely available database search and its articles.

The 2015 WHO State of inequality report indicates that women who are poor, least educated, and who reside in rural areas have lower coverage of health interventions and worse health outcomes than more advantaged women [46]. Interventions among neonates and infants that promote healthy developmental outcomes could assist to address health equity. Newborn/infant massage is a relatively simple and accessible intervention across a range of settings. Provided the necessary training and support is available, this intervention may increase health equity. However, more evidence is required on the effectiveness of massage including various aspects such as the use of oils/emollients for massage, type of provider, frequency, duration, length, and technique to guide optimal and safe practices. Adequately powered trials should be conducted in future, focusing on short-term and long-term meaningful outcomes and addressing the methodological fallacies of the existing studies. These outcomes may include mortality, growth and neurodevelopment outcomes as well as adverse events like injuries, slippages etc. Some of the important fallacies can be addressed by ensuring adequate randomization, consistency of the intervention (technique, massage provider, dose and duration), optimum follow up with minimal attrition, and blinded outcome assessment by skilled staff using standard measurement tools.

## CONCLUSIONS

Whole-body massage may improve the body length in term healthy newborns. The evidence on the effects of infant massage on growth, behavior, and neurodevelopment outcomes is uncertain. There is a need for further well-designed trials to assess the impact of whole-body massage on short- and longer-term outcomes in term healthy newborns.

## Additional material:


Online Supplementary Document
