# Supplementary material for: Effect of whole-body massage on growth and neurodevelopment in term healthy newborns: A systematic review
Source: J Glob Health. 2022 Oct 18;12:12005. doi: 10.7189/jogh.12.12005 (PMC9577283; doi:10.7189/jogh.12.12005)
Supplement: Online Supplementary Document [file jogh-12-12005-s001.pdf]

ONLINE SUPPLEMENTARY DOCUMENT

Effect of Whole-Body Massage on Growth and Neurodevelopment in Term Healthy Newborns: A Systematic Review

Appendix S1. Search strategy

We used the following search terms for MEDLINE: (Newborn OR infant OR neonat*) AND (massage OR therapeutic touch OR tactile stimulation OR pressure stroking). Similar terms were used for searching the other databases: Cochrane Central Register of Controlled Trials, EMBASE, and CINAHL.

Appendix S2. Risk of bias in included studies

Thirty of the 31 trials were judged to be at high risk of bias, with most studies being at high risk of bias in the randomization process. Twenty-five studies were at high risk of bias for randomization, either due to non-reporting of random sequence generation or allocation concealment or owing to their quasi-randomized design. While 19 trials did not specify any details of randomization (either sequence generation or allocation concealment), seven trials (Chen 2011, Ding 2005, Gultom 2019, Gurol 2012, Field 1996, Zhai 2001, and Zhu 2010) used quasi-randomized design. All studies were either judged to be at 'high risk' (9 trials) or 'some concerns' (22 trials) for deviation from intended interventions. This was due to the lack of blinding of the parents or trainers and lack of any specified mechanism to monitor adherence in any of the studies. 21 trials were judged to be at high risk of bias for missing outcome data. Five trials (Chen 2011, Inal 2012, Jing 2007, Koniak-griffin 1988, and Seyyedrasooli 2014) had an unacceptably high rate of attrition at follow-up (>30% of the study population). 16 studies did not report any details of missing outcomes or any efforts to prevent loss to follow up. 18 studies were judged to be at high risk of bias for outcome measurement because these studies did not report any details of outcome assessment: blinding of assessors, outcome assessment methods, etc. Five trials were judged to be at 'high risk' for bias in selective reporting. These trials (Ke 2001, Liu DY 2005, Maimaiti 2007, Sun 2004, and Xua 2004) did not report all of the pre-specified outcomes or reported them incompletely. A summary of the risk of bias assessment is depicted in Figure S1 and Figure S2.

Figure S1. Risk of bias "traffic light" plots: review authors' judgments about each risk of bias item for each included study

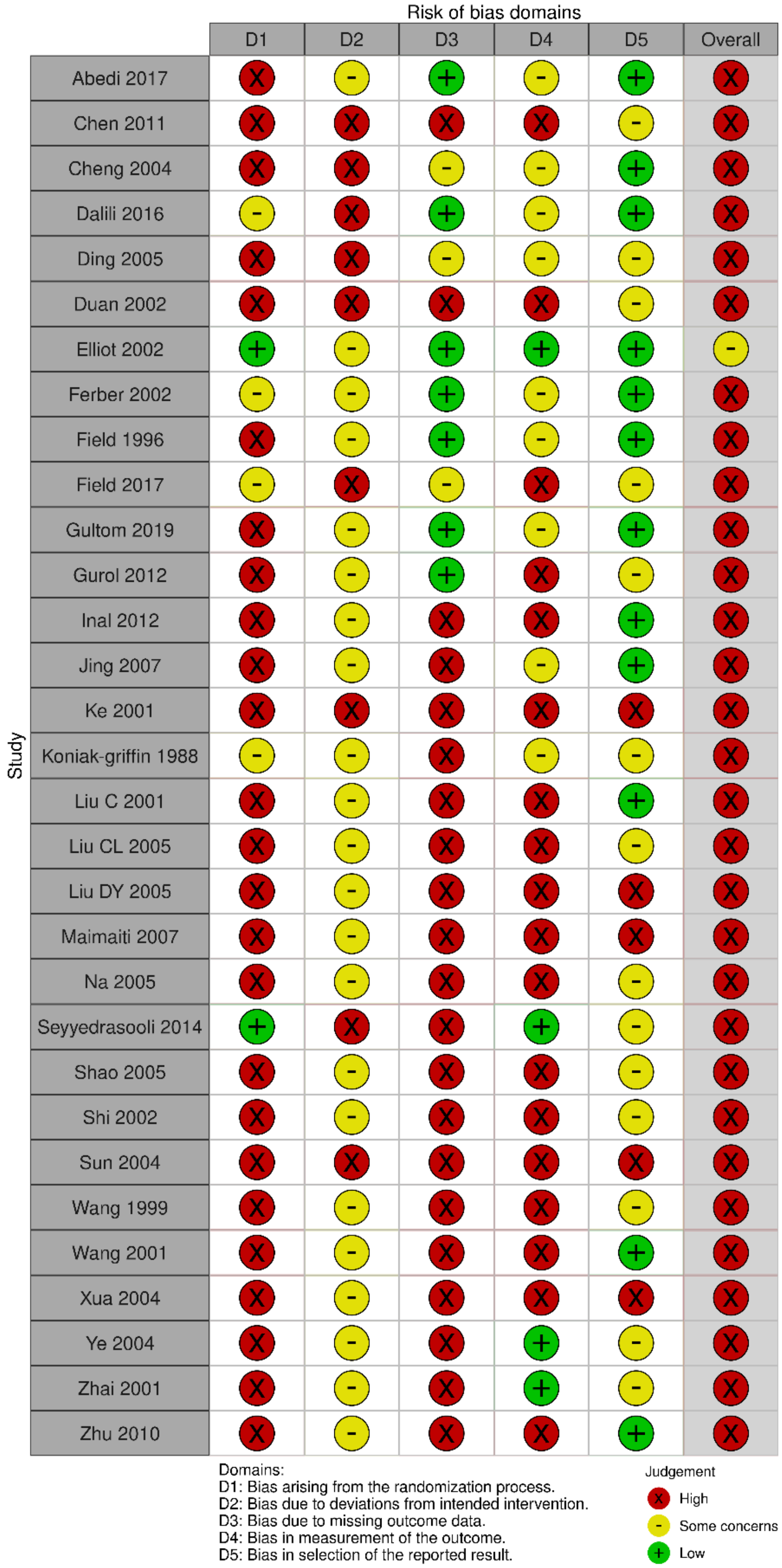

Figure S2. Risk of bias "weighted bar plots": review authors' judgments about each risk of bias item presented as percentages across all included studies

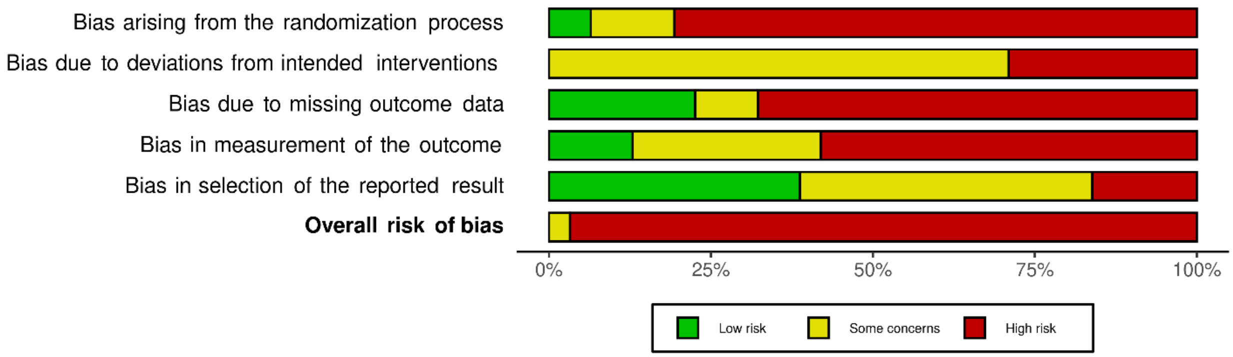

Figure S3 Panel A. Funnel plot for comparison: Massage vs. no massage, Outcome: Infant weight at the end of intervention period

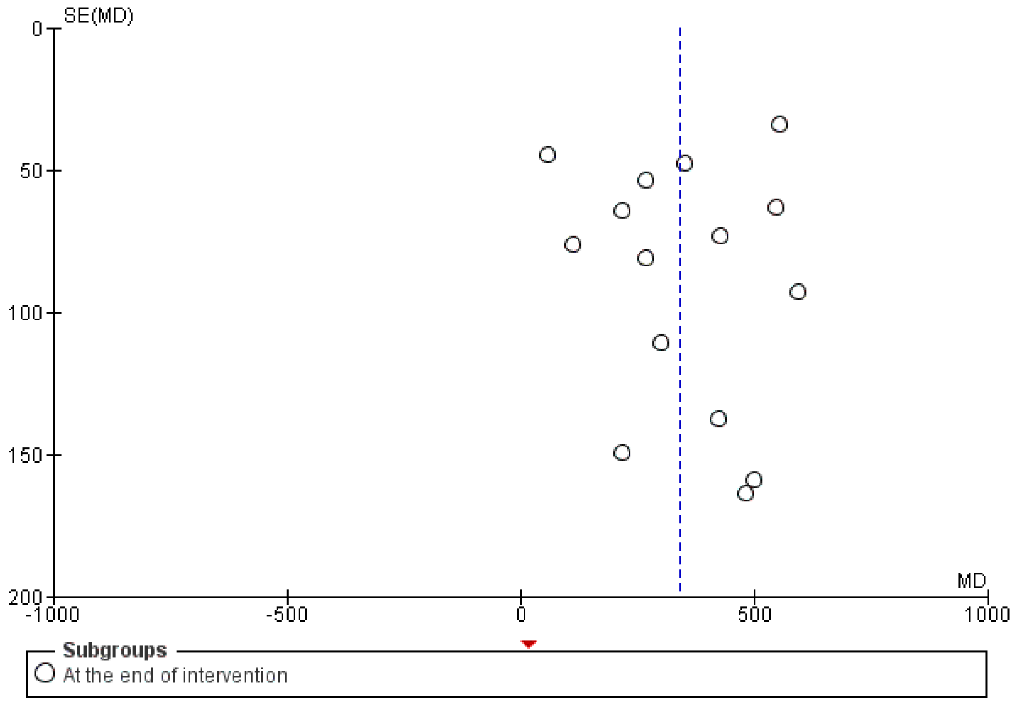

Figure S3 Panel B. Funnel plot for comparison: Massage vs. no massage, Outcome: Infant length at the end of intervention period

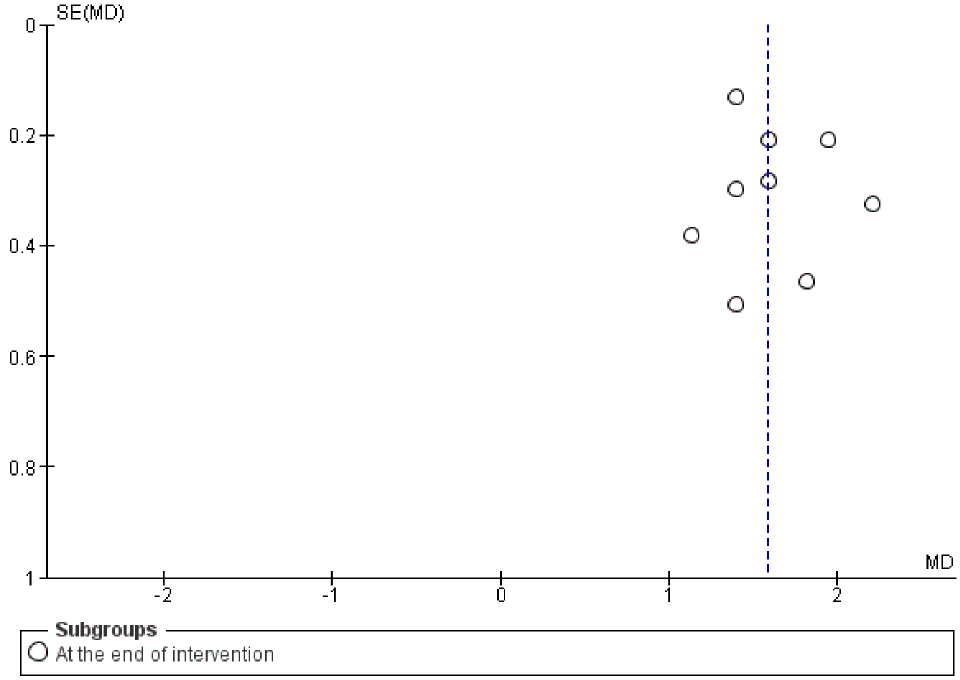

Figure S4 Panel A. Sensitivity analysis for comparison: Massage vs. no massage, Outcome: Infant weight at the end of intervention period

	Experimental			Control				Mean Difference
Study or Subgroup	Mean [g]	SD [g]	Total	Mean [g]	SD [g]	Total	Weight	IV, Fixed, 95% CI
**1.1.1 At the end of intervention**								
Abedi 2017	4,463.326	319	30	4,351	270	30	15.6%	112.33 [-37.22, 261.88]
Cheng 2004	5,198	430	50	4,932	381	50	13.7%	266.00 [106.76, 425.24]
Ding 2005	5,506	442	150	5,154.4	387.4	150	39.4%	351.60 [257.54, 445.66]
Field 1996	7,393.56	1,360.78	20	6,985.32	1,360.78	20	0.5%	408.24 [-435.16, 1251.64]
Gultom 2019	3,806	552.79	17	3,588.24	270.65	17	4.1%	217.76 [-74.82, 510.34]
Jing 2007	8,650	1,090	90	8,150	1,040	90	3.6%	500.00 [188.75, 811.25]
Koniak-Griffin 1988	6,878.58	855.5	20	6,862	871.93	21	1.2%	16.58 [-512.24, 545.40]
Ye 2004	5,391	345.63	50	5,174	297.14	50	21.8%	217.00 [90.66, 343.34]
**Subtotal (95% CI)**			**427**			**428**	**100.0%**	**269.12 [210.07, 328.17]**
Heterogeneity: Chi² = 11.04, df = 7 (P = 0.14); I² = 37%								
Test for overall effect: Z = 8.93 (P < 0.00001)								

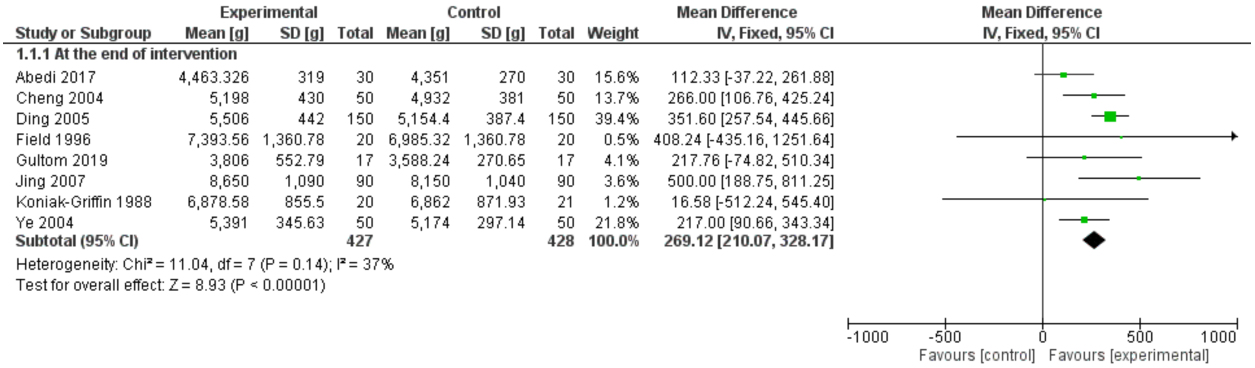

Figure S4 Panel B. Sensitivity analysis for comparison: Massage vs. no massage, Outcome: Infant length at the end of intervention period

	Experimental			Control				Mean Difference
Study or Subgroup	Mean [cm]	SD [cm]	Total	Mean [cm]	SD [cm]	Total	Weight	IV, Fixed, 95% CI
**1.2.1 At the end of intervention**								
Abedi 2017	54.283	1.98	30	52.463	1.6	30	13.8%	1.82 [0.91, 2.73]
Cheng 2004	57.45	1.38	50	55.86	1.44	50	37.5%	1.59 [1.04, 2.14]
Gultom 2019	50.75	1.01	17	48.54	0.88	17	28.3%	2.21 [1.57, 2.85]
Jing 2007	68.68	2.29	90	67.55	2.81	90	20.4%	1.13 [0.38, 1.88]
**Subtotal (95% CI)**			**187**			**187**	**100.0%**	**1.70 [1.36, 2.04]**
Heterogeneity: Chi² = 4.91, df = 3 (P = 0.18); I² = 39%								
Test for overall effect: Z = 9.86 (P < 0.00001)								
Test for subgroup differences: Not applicable								

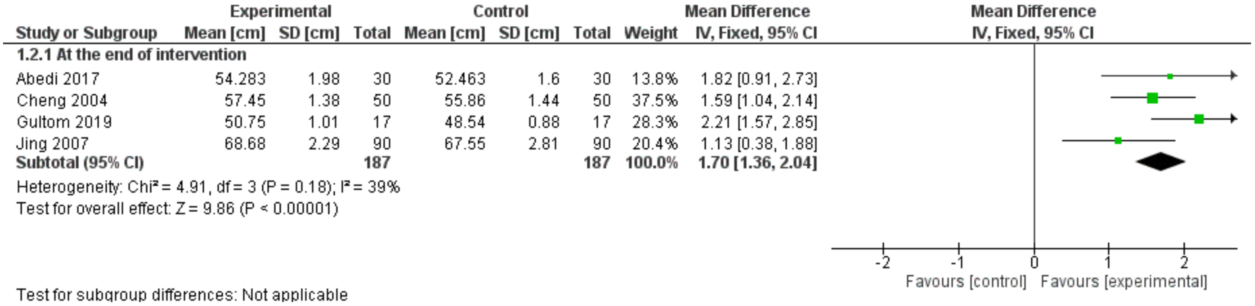

Figure S4 Panel C. Sensitivity analysis for comparison: Massage vs. no massage, Outcome: Infant head circumference at the end of intervention period

	Experimental			Control				Mean Difference
Study or Subgroup	Mean [cm]	SD [cm]	Total	Mean [cm]	SD [cm]	Total	Weight	IV, Fixed, 95% CI
**1.3.1 At the end of intervention**								
Abedi 2017	37.29	0.8	30	36.816	0.82	30	62.3%	0.47 [0.06, 0.88]
Cheng 2004	39.16	1.22	50	38.24	1.46	50	37.7%	0.92 [0.39, 1.45]
**Subtotal (95% CI)**			**80**			**80**	**100.0%**	**0.64 [0.32, 0.97]**
Heterogeneity: Chi² = 1.71, df = 1 (P = 0.19); I² = 42%								
Test for overall effect: Z = 3.89 (P = 0.0001)								
Test for subgroup differences: Not applicable								

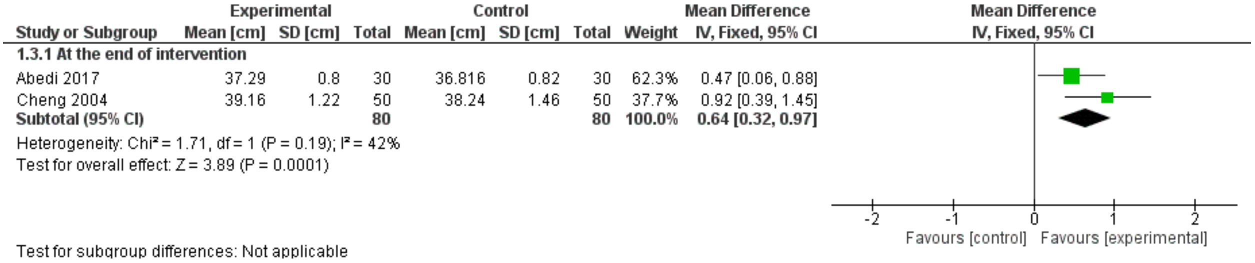

Table S1. GRADE table: Whole-body massage compared with no massage

Certainty assessment							№ of patients		Effect		Certainty (GRADE)	Importance
**№ of studies**	**Study design**	**Risk of bias**	**Inconsistency**	**Indirectness**	**Imprecision**	**Other considerations**	**Whole-body massage**	**No massage**	**Relative (95% CI)**	**Absolute (95% CI)**		
**Weight – end of intervention period (grams)**												
17	randomised trials	very serious [a]	serious [b]	not serious	not serious	none	1089	1093	-	MD **340.30 higher** (239.81 higher to 440.79 higher)	⨁◯◯◯ VERY LOW	CRITICAL
**Weight – follow-up at 8–12 months (grams)**												
2	randomized trials	very serious [a]	not serious	not serious	serious [c]	none	74	83	-	MD **455.07 higher** (86.33 higher to 823.8 higher)	⨁◯◯◯ VERY LOW	CRITICAL
**Length – end of intervention period (centimetres)**												
9	randomised trials	very serious [a]	not serious	not serious	not serious	none	647	647	-	MD **1.58 higher** (1.42 higher to 1.74 higher)	⨁⨁◯◯ LOW	CRITICAL
**Length – follow-up at 12 months (centimetres)**												
1	randomized trials	very serious [a]	not serious	not serious	very serious [c,d]	none	54	62	-	MD **0.71 higher** (0.15 lower to 1.57 higher)	⨁◯◯◯ VERY LOW	CRITICAL
**Head circumference – end of intervention period (centimetres)**												
6	randomized trials	very serious [a]	serious [b]	not serious	not serious	none	500	500	-	MD **0.85 higher** (0.57 higher to 1.14 higher)	⨁◯◯◯ VERY LOW	CRITICAL
**Head circumference – follow-up at six months (centimetres)**												
1	randomized trials	very serious [a]	not serious	not serious	serious [c]	none	55	60	-	MD **1.31 higher** (0.55 higher to 2.07 higher)	⨁◯◯◯ VERY LOW	CRITICAL
**Bilirubin levels at four days (mmol/L)**												
4	randomized trials	very serious [a]	not serious	not serious	serious [c]	none	168	177	-	MD **31.75 lower** (40.05 lower to 23.46 lower)	⨁◯◯◯ VERY LOW	CRITICAL

Certainty assessment							№ of patients		Effect		Certainty (GRADE)	Importance
№ of studies	Study design	Risk of bias	Inconsistency	Indirectness	Imprecision	Other considerations	Whole-body massage	No massage	Relative (95% CI)	Absolute (95% CI)		
**Crying or fussing time – end of intervention (hours/day)**												
3	randomised trials	very serious [a]	not serious	not serious	serious [c]	none	136	135	-	MD **0.36 lower** (0.16 lower to 0.56 lower)	⨁◯◯◯ VERY LOW	CRITICAL
**Crying or fussing time - follow-up 6 months (hours/day)**												
1	randomised trials	very serious [a]	not serious	not serious	serious [c]	none	61	63	-	MD **0.15 lower** (0.01 lower to 0.29 lower)	⨁◯◯◯ VERY LOW	CRITICAL
**Sleep duration over 24 hour period – end of intervention (hours/day)**												
3	randomized trials	very serious [a]	serious [b]	not serious	not serious	none	266	268	-	MD **0.62 higher** (0.12 higher to 1.12 higher)	⨁◯◯◯ VERY LOW	CRITICAL
**Sleep duration over 24 hour period – follow-up at six months (hours/day)**												
1	randomized trials	very serious [a]	not serious	not serious	very serious [c,d]	none	61	63	-	MD **0.08 higher** (0.48 lower to 0.64 higher)	⨁◯◯◯ VERY LOW	CRITICAL
**Psychomotor Development Indices (PDI) meta-analysis post-intervention – end of intervention period**												
3	randomized trials	very serious [a]	not serious	not serious	serious [c]	none	234	154	-	SMD **0.39 higher** (0.6 higher to 0.18 higher)	⨁◯◯◯ VERY LOW	CRITICAL
**Psychomotor Development Indices (PDI) meta-analysis post-intervention – follow-up at 24 months**												
1	randomized trials	very serious [a]	not serious	not serious	very serious [c,d]	none	20	21	-	SMD 7.52 higher**0** (1.49 lower to 16.53 higher)	⨁◯◯◯ VERY LOW	CRITICAL
**Mental Development Indices (MDI) meta-analysis post-intervention – end of intervention period**												
3	randomized trials	very serious [a]	serious [b]	not serious	very serious [c,d]	none	234	154	-	SMD **0.29 higher** (0.18 lower to 0.77 higher)	⨁◯◯◯ VERY LOW	CRITICAL

Certainty assessment							№ of patients		Effect		Certainty (GRADE)	Importance
№ of studies	Study design	Risk of bias	Inconsistency	Indirectness	Imprecision	Other considerations	Whole-body massage	No massage	Relative (95% CI)	Absolute (95% CI)		
**Mental Development Indices (MDI) meta-analysis – follow-up at 24 months**												
1	randomized trials	very serious [a]	not serious	not serious	very serious [c,d]	none	20	21	-	SMD **8.59 higher** (1.62 lower to 18.80 higher)	⊕◯◯◯ VERY LOW	CRITICAL
**Gross motor development at end of intervention (Gesell development quotient/Capital Institute mental checklist)**												
2	randomized trials	very serious [a]	not serious	not serious	serious [c]	none	117	120	-	SMD **0.44 higher** (0.18 higher to 0.7 higher)	⊕◯◯◯ VERY LOW	CRITICAL
**Fine motor development at end of intervention (Gesell development quotient/Capital Institute mental checklist)**												
2	randomized trials	very serious [a]	not serious	not serious	serious [c]	none	117	120	-	SMD **0.61 higher** (0.35 higher to 0.87 higher)	⊕◯◯◯ VERY LOW	CRITICAL
**Language at end of intervention (Gesell development quotient/Capital Institute mental checklist)**												
2	randomized trials	very serious [a]	serious [b]	not serious	very serious [c,d]	none	117	120	-	SMD **0.82 higher** (0.03 lower to 1.67 higher)	⊕◯◯◯ VERY LOW	CRITICAL
**Personal-social behaviour at end of intervention (Gesell development quotient/Capital Institute mental checklist)**												
2	randomized trials	very serious [a]	serious [b]	not serious	serious [c]	none	117	120	-	SMD **0.9 higher** (0.18 higher to 1.61 higher)	⊕◯◯◯ VERY LOW	CRITICAL
**Gross motor development at 12 months (Gesell development quotient)**												
1	randomized trials	very serious [a]	not serious	not serious	very serious [c,d]	none	54	62	-	MD **2.85 higher** (2.48 lower to 8.18 higher)	⊕◯◯◯ VERY LOW	CRITICAL
**Fine motor development at 12 months (Gesell development quotient)**												
1	randomized trials	very serious [a]	not serious	not serious	serious [c]	none	54	62	-	MD **8.12 higher** (4.57 higher to 11.67 higher)	⊕◯◯◯ VERY LOW	CRITICAL
**Language at 12 months (Gesell development quotient)**												

Certainty assessment							№ of patients		Effect		Certainty (GRADE)	Importance
№ of studies	Study design	Risk of bias	Inconsistency	Indirectness	Imprecision	Other considerations	Whole-body massage	No massage	Relative (95% CI)	Absolute (95% CI)		
1	randomized trials	very serious [a]	not serious	not serious	serious [c]	none	54	62	-	MD **7.9 higher** (4.1 higher to 11.7 higher)	⨁◯◯◯ VERY LOW	CRITICAL
**Personal-social behaviour at 12 months (Gesell development quotient)**												
1	randomized trials	very serious [a]	not serious	not serious	serious [c]	none	54	62	-	MD **6.19 higher** (2.55 higher to 9.83 higher)	⨁◯◯◯ VERY LOW	CRITICAL
**Maternal Attachment Inventory score**												
1	randomized trials	very serious [a]	not serious	not serious	serious [c]	none	57	60	-	MD **5.77 higher** (0.95 higher to 10.59 higher)	⨁◯◯◯ VERY LOW	CRITICAL

CI: confidence interval, MD: mean difference; SMD: standardized mean difference
a. The pooled effect provided by studies at high risk of bias.
b. Severe, unexplained, heterogeneity ($I^2 \geq 60\%$ or p-value < 0.05).
c. Less than 400 participants.
d. Wide confidence interval crossing the line of no effect.
